# On the Use of *t*-Distributed Stochastic Neighbor Embedding for Data Visualization and Classification of Individuals with Parkinson's Disease

**DOI:** 10.1155/2018/8019232

**Published:** 2018-11-04

**Authors:** Fábio Henrique M. Oliveira, Alessandro R. P. Machado, Adriano O. Andrade

**Affiliations:** ^1^Centre for Innovation and Technology Assessment in Health, Postgraduate Program in Electrical and Biomedical Engineering, Faculty of Electrical Engineering, Federal University of Uberlândia, Uberlândia, Brazil; ^2^Federal Institute of Science and Technology–Campus Brasília, Brasília, Brazil

## Abstract

Parkinson's disease (PD) is a neurodegenerative disorder that remains incurable. The available treatments for the disorder include pharmacologic therapies and deep brain stimulation (DBS). These approaches may cause distinct side effects and motor responses. This work presents the application of *t*-distributed stochastic neighbor embedding (*t*-SNE), which is a machine learning algorithm for nonlinear dimensionality reduction and data visualization, for the problem of discriminating neurologically healthy individuals from those suffering from PD (treated with levodopa and DBS). Furthermore, the assessment of classification methods is presented. Inertial and electromyographic data were collected while the subjects executed a sequence of four motor tasks. The results were focused on the comparison of the classification performance of a support vector machine (SVM) while discriminating two-dimensional feature sets estimated from Principal Component Analysis (PCA), Sammon's mapping, and *t*-SNE. The results showed visual and statistical differences for all three investigated groups. Classification accuracy for PCA, Sammon's mapping, and *t*-SNE was, respectively, 73.5%, 78.6%, and 96.9% for the training set and 67.8%, 74.1%, and 76.6% for the test set. The possibility of discriminating healthy individuals from those with PD treated with levodopa and DBS highlights the fact that each treatment method produces distinct motor behavior. The scatter plots resulting from *t*-SNE could be used in the clinical practice as an objective tool for measuring the discrepancy between normal and abnormal motor behaviors, being thus useful for the adjustment of treatments and the follow-up of the disorder.

## 1. Introduction

Parkinson's disease (PD) is one of the most common neurodegenerative disorders, which remains incurable and affects approximately 3% of the population over 65 years of age [1]. Patients affected by PD may have resting tremor (oscillatory movement), bradykinesia (slowness of movement), rigidity (increased muscular tone), and impairment in their ability to initiate and sustain movements [1–4]. The PD incidence ratio is expected to increase as people live longer; thus, aging is an important risk factor in PD [5].

The disease diagnosis is usually a critical point. It is estimated that currently 20% of patients are not correctly diagnosed [6]. According to a review [7] which evaluated the accuracy of clinical diagnosis of PD from 1988 to 2014, the correct diagnosis is crucial for prognostic and therapeutic reasons and clinical, pharmacologic, and epidemiologic studies as well. Despite advances in neuroimaging and genetics, the diagnosis of PD remains primarily clinical [7].

Epidemiology is the study of how often diseases occur in different groups of people and why [8]. The quantitative element of epidemiological studies is directly related to the diagnosis of a disease, in this case, PD. If a subject is misdiagnosed with PD, this affects the statistics of epidemiological studies and vice versa. Furthermore, this information is used in many types of research.

A number of rating scales are used for the evaluation of motor impairment and disability in patients with PD. The Unified Parkinson's Disease Rating Scale (UPDRS) is the most well-established subjective scale for assessing disability and impairment [9, 10]. Such scale is composed of four parts: Part I (nonmotor experiences of daily living), Part II (motor experiences of daily living), Part III (motor examination), and Part IV (motor complications). There are a number of alternative rating scales that are used for the evaluation of motor impairment and disability in patients with PD, but these scales have not been fully evaluated for validity and reliability [2]. Due to these subjective methods that are currently used and the need for improving the diagnosis and treatment efficacy, studies must be performed to provide feedback for neurologists during clinical evaluation of patients, reducing the time and effort required to achieve optimal outcomes and improving the treatment.

Some of the PD symptoms can be reduced with pharmacological and/or surgical intervention, and the lifespan of the patients can consequently be extended. The drug levodopa (LD) is one of the most effective and widely used for PD treatment [11, 12]. Surgical interventions, such as pallidotomy (ablation/lesioning) and Deep Brain Stimulation (DBS), have also established efficacy in the treatment of PD [13].

DBS therapy delivers electrical stimulation to areas in the brain, alleviating PD motor symptoms. The patient is a candidate for this type of therapy if the symptoms do not respond effectively to levodopa [14].

Regarding the differences between DBS and medication-based treatments, several studies [14–18] show comparative results. Most of these studies assess PD patients treated with DBS versus medication employing subjective scales to evaluate each method. They found that DBS provided better outcomes in motor activity. Furthermore, the authors highlighted that the group which received neurostimulation is more susceptible to serious adverse effects, including fatal cerebral hemorrhage.

An extensive review suggests that the major surgery-related risk is intracranial hemorrhage and the overall incidence of hemorrhage was 5.0%, with symptomatic hemorrhage occurring in 2.1% of patients and hemorrhage resulting in permanent neurological deficit or death in 1.1% [19].

Additionally, objective approaches to evaluate DBS and medication-based treatments are not well explored. Machado et al. [20] conducted a study to compare, in an objective way, three groups of subjects (i.e., PD patients treated with DBS and levodopa, PD patients treated only with levodopa and healthy subjects). Each subject performed a set of static and dynamic tasks. The aim of the study was to introduce a method for automatic classification among these groups in a high-dimensional space.

Although several studies investigated and compared DBS versus medication-based treatments by means of rating scales (e.g., UPDRS) until now, just a few studies used objective methods for comparing and visualizing the possible differences between patients treated differently. As reported in [14, 18], subjects treated with DBS plus medication presented better results than medication treatment alone in terms of motor behavior. In this way, an automatic classification of these groups could be able to compare them and show if patients treated with DBS present the expected improvements or/and if they have the DBS parameters correctly set.

A relevant area for data visualization is dimensionality reduction (DR). DR focuses on keeping data relationship from high-dimensional (e.g., original data) to low-dimensional (e.g., reduced data) spaces. In addition, DR methods are used to simplify data visualization, making it easier for human evaluation. Data visualization is an important application of DR. It is the study of the visual representation of data through graphical representations, and it is effective in exploratory data analysis [21, 22].

DR algorithms can be divided into different categories based on different criteria, e.g., linear and nonlinear dimensionality reduction algorithms. Classically, the problem of dimension reduction and data representation has been approached by applying linear transformations such as the well-known principal component analysis (PCA) [23, 24]. Those linear techniques focus on keeping the low-dimensional representations of dissimilar data points far apart. However, PCA is not capable of representing higher order, nonlinear, and local structure in the data. In the last decades, some nonlinear DR algorithms have been proposed to deal with complex nonlinear data.

Many nonlinear and linear DR methods are reported in the literature [25, 26]. In this paper, three of these methods are assessed: PCA [23], Sammon's mapping [27], and *t*-distributed stochastic neighbor embedding (*t*-SNE) [28]. Features in a low-dimensional space are classified based on their ability to discriminate neurologically healthy individuals, individuals suffering from PD treated with levodopa and individuals suffering from PD treated with DBS.

## 2. Methods

### 2.1. Participants and Data Collection

This study was conducted in the Federal University of Uberlândia (UFU), Uberlândia, Brazil, and at the University of California, Los Angeles (UCLA), USA. Both institutions provided ethical approval for the experimental procedures (CAAE 07075413.6.0000.5152; UCLA IRB 14-001491). A complete description of the procedure employed for data collection is available in [20].

The dataset consists of motor task measurements collected from 38 subjects. The subjects were divided into the following groups: neurologically healthy individuals (*S*_H_ = 10), individuals suffering from PD treated with levodopa (*S*_PD_ = 16), and individuals suffering from PD treated with DBS (*S*_DBS_ = 12). All the subjects with PD that participated in this study were rated as 2 (i.e., bilateral or midline involvement without impairment of balance) or 3 (i.e., bilateral disease: mild-to-moderate disability with impaired postural reflexes; physically independent) by using Hoehn and Yahr scale [29].

**Figure 1:**
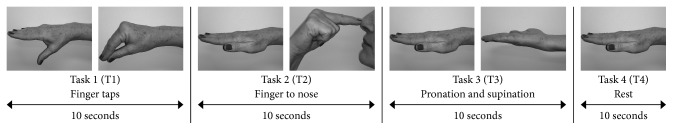
Basic sequence of executed tasks.

The dataset used in this study resulted from four motor tasks depicted in Figure 1, performed by the volunteers: finger taps (Task 1 - T1), finger to nose (Task 2 - T2), supination and pronation (Task 3 - T3) and rest (Task 4 - T4).

Each subject executed the sequence of four tasks depicted in Figure 1 five times. At least 30 s was allowed for rest after the end of the execution of each sequence (from tasks 1 to 4).

During the execution of the tasks, two sets of three-axial inertial sensors (i.e., accelerometer, gyroscope, and magnetometer), weighing 1 g each, were positioned on the dorsal surface of hand and forearm. Two pairs of disposable electromyographic (EMG) sensors were placed on the muscles flexor and extensor of the forearm. Both inertial and the envelope of EMG signals were digitized at 50 Hz.

**Figure 2:**
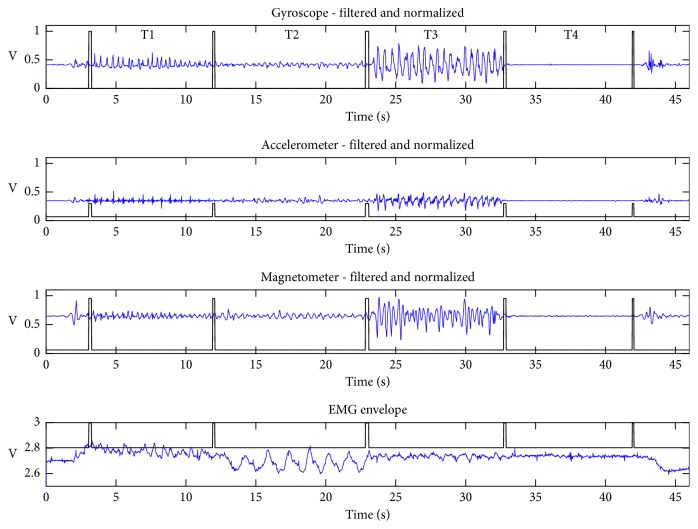
Typical example of preprocessed signals. Results of the application of the windowing and filtering steps described in [20]. The distinct tasks (T1, T2, T3, and T4) are separated by pulses.

Figure 2 illustrates typical waveforms of resultant components (i.e., a combination of *x*, y, and *z* coordinates) for the inertial sensors and the signal envelope for the electromyographic activity. The periods of the sequence of executed tasks (T1, T2, T3, and T4) are delimited by rectangular windows, indicating the beginning and end of each task.

Since each subject repeated each task five times, it was computed the coefficient of variation (CV) [30] to estimate the ratio of the standard deviation to the mean among the repetitions. For the reproducibility perspective, CV value can be used as one parameter to guide other studies in the reproduction of the experiment results.

On average, Table 1 shows the coefficients of variation for the subjects per group. *S*_H_ presented lower CV value among the three groups indicating that the subjects from this group do not vary in terms of the motor pattern as much as subjects from *S*_PD_ and *S*_DBS_ groups. On the other hand, subjects from *S*_PD_ and *S*_DBS_ groups vary more, which is expected once they suffer from PD presenting different motor patterns according to their physiological conditions (e.g., under medication and anxiety).

### 2.2. Steps for Data Processing

**Figure 3:**
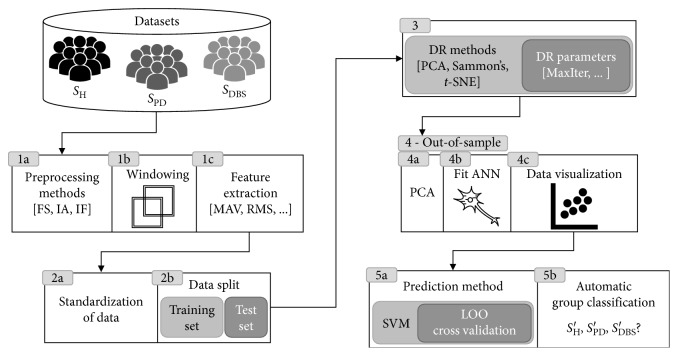
Diagram depicting the main signal processing steps.

Focusing on data visualization and the discrimination between healthy subjects from those suffering from PD, the present study assesses features estimated from data projection techniques (PCA, Sammon's mapping, and *t*-SNE) classified by a support vector machine (SVM) classifier. The main steps of this study are shown in Figure 3.

The extracted features were standardized (step 2a in Figure 3) and then split into training and test sets (step 2b in Figure 3). The high-dimensional feature vectors of the training set were submitted to dimension reduction (step 3 in Figure 3). The corresponding low-dimensional map point for the test set was produced by means of an out-of-sample extension technique (step 4 in Figure 3). This step was accomplished by using an artificial neural network (ANN).

Feature reduction was followed by supervised learning and classification, which was achieved through SVM [31] (step 5 in Figure 3). These steps aim to evaluate the DR techniques in order to explore the PD motor task data. Each used method is described in detail in the following subsections.

### 2.3. Feature Extraction

Feature extraction was performed over the filtered signals (FS), the instantaneous amplitude (IA), and the instantaneous frequency (IF), estimated from the Hilbert transform [32], as pointed out in the step 1c of Figure 3. The following features, which are fully described in Table 1 of [33, 34], were estimated: mean absolute value (MAV), root mean squared (RMS), global maximum (PEAK), mean of the absolute values of the second differences of the normalized signal (MAVSDN), mean of the absolute values of the second differences (MAVSD), mean of the absolute values of the first differences of the normalized signal (MAVFDN), mean of the absolute values of the first differences of the signal (MAVFD), interquartile range of the signal (INTERQ_RANGE), difference between the maximum and minimum values of a signal (RANGE), standard deviation (STD), variance (VAR), and approximate entropy.

For each method (i.e., FS, IA, and IF), a feature matrix was created containing the features extracted from all sensors. In addition, it was analyzed the combination of features estimated from each method: FS-IA, FS-IF, IA-IF, and FS-IA-IF. The aim was to identify which combination could provide the best discrimination results. The preprocessing methods (step 1a in Figure 3) are fully described in [20].

### 2.4. Data Standardization and Splitting

Since we have data from different sensors (i.e., accelerometer, gyroscope, magnetometer, and electromyography) which are on different scales, it is common to standardize the data. Thus, the features were standardized by using the zscore method (step 2a in Figure 3),

(1) $$\begin{array}{c} z=\frac{x-\mu }{\sigma }, \end{array}$$

where *x* is the feature to be standardized, *μ* is the mean of the feature including all samples, and *σ* is the standard deviation of that feature. The standardized feature vectors were then separated randomly into training and test sets (step 2b in Figure 3) comprising 90% and 10%, respectively, of the data from each group of subjects (*S*_H_, *S*_PD_, and *S*_DBS_) before proceeding. A strict separation between training and test sets is crucial for a more real and reliable evaluation of the automated classification task. This is an improvement while compared to the study described in [20], where the dimension reduction step was applied to the entire dataset prior to machine learning.

### 2.5. Unsupervised Dimension Reduction Analysis

In this work, three unsupervised DR methods were evaluated (step 3 in Figure 3). The first one was the linear feature reduction PCA [23, 24]. The second was Sammon's mapping, one of the first nonlinear mapping algorithms for analysis of multivariate data [27].

The third, also a nonlinear mapping technique, was *t*-SNE of van der Maaten and Hinton [28]. *t*-SNE is an improved variation of the stochastic neighbor embedding (SNE) [35]. *t*-SNE tries to place a point from high-dimensional space in a low-dimensional one so as to preserve neighborhood identity. The SNE algorithm converts Euclidean distances between high-dimensional data points into conditional probabilities representing similarities; closer data points mean high similarity.

The similarity of data point *x*_*j*_ to data point *x*_*i*_ is represented by the conditional probability *p*_*j*|*i*_. These similarities express the probability that *x*_*i*_ would select *x*_*j*_ as its neighbor. For the low-dimensional counterparts *y*_*i*_ and *y*_*j*_ of the high-dimensional data points *x*_*i*_ and *x*_*j*_, it is computed a similar conditional probability denoted by *q*_*j*|*i*_.

Once conditional probability distributions are calculated for the data points in both the high- and low-dimensional representations, the goal of the algorithm is to minimize the mismatch between the two. The cost function (Equation (2)) which should be minimized is the sum of Kullback–Leibler (KL) divergences over all points using a gradient descent method:

(2) $$\begin{array}{c} E=\underset{i}{\sum }KL\left(P_{i}||Q_{i}\right)=\underset{i}{\sum }\underset{j}{\sum }p_{j\left|i\right.}\log\frac{p_{j\left|i\right.}}{q_{j\left|i\right.}}, \end{array}$$

in which *P*_*i*_ represents the conditional probability distribution over all data points given a data point *x*_*i*_ and *Q*_*i*_ represents the conditional probability distribution over all other map points given map point *y*_*i*_.

*t*-SNE improves SNE in two points [28]: (1) by using a symmetrized version of the SNE cost function with simpler gradients and (2) by applying Student's *t*-distribution rather than a Gaussian to compute the similarity between two points in the low-dimensional space.

For each of employed DR method, the high-dimensional data (i.e., all features estimated from EMG, accelerometer, gyroscope, and magnetometer sensors) were reduced to a two-dimensional space. Data projections were carried out for each scenario or experiment (see Section 2.5.1 for more details) and then a scatter plot of the obtained projection was generated (step 4c in Figure 3) so that possible differences among the studied groups could be visualized.

#### 2.5.1. Parameter Setting

Sammon's mapping and *t*-SNE have several free parameters, such as the number of iterations for which the cost function optimization is processed and the learning rate used in the gradient descent method. In addition, *t*-SNE has perplexity parameter, which can be defined as a smooth measure of the effective number of neighbors.

**Figure 4:**
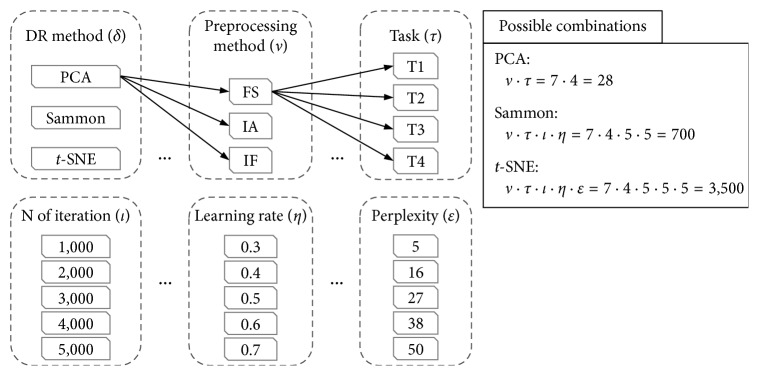
Parameter settings of the experiments. Note that for the preprocessing methods we explore all possible combinations between them (i.e., FS, IF, IA, FS-IF, FS-IA, IF-IA, and FS-IF-IA).

In our experiments, we did an *exhaustive* search in order to evaluate the influence of each DR parameter in the quality of the generated maps. All the parameter settings are shown in Figure 4.

Each DR method was evaluated across some experiments without repetition (same combination more than once), which are composed by different parameter settings (as shown in Figure 4); for example, PCA experiments are arranged by the combination of preprocessing methods (*v*) and tasks (*τ*), resulting in 28 experiments. Following, with a total of 700 experiments is Sammon's mapping by the combination of *v*, *τ*, number of iterations (*l*), and learning rate (*η*). Lastly, *t*-SNE experiments combine all parameters depicted in Figure 4, which sums 3,500 experiments.

For each setup shown in Figure 4, the procedure was (1) execute DR method; (2) execute the out-of-sample process; (3) train and test the SVM classifier; and (4) compute performance indices in order to evaluate the parameters setup.

### 2.6. Out-of-Sample Extension

A plenty of nonlinear DR methods only map a given finite set of data points to low-dimension, not providing a built-in way to map new data points to the corresponding low-dimensional representation. Sammon's mapping and *t*-SNE fall into this category of DR methods. The training set of high-dimensional data *x*_*i*_ and their corresponding mapped low-dimensional representation *y*_*i*_ was used to train a feedforward neural network with weights *w*, which act as a mapping function *f*∶*x*_*i*_ → *y*_*i*_ in which for each *x*_*i*_, we have a *y*_*i*_ to determine the low-dimensional representation of the test set (step 4b in Figure 3).

Before proceeding to use an ANN, the high-dimensional training set passes through PCA by preserving 90% of the total variance of the data (step 4a in Figure 3). This step avoids the curse of dimensionality [36] and speeds up ANN training. Bayesian regularization backpropagation [37] was the training function used to update *w* and bias values.

The analysis of the lower dimensional data was performed by means of the evaluation of classification results.

### 2.7. Classification Analysis

In order to evaluate the DR techniques, a supervised machine learning classifier, support vector machine (SVM), was employed for data classification (step 5 in Figure 3). Once trained, the model was cross-validated using a leave-one-out (LOO) method and the cross validation loss of the model was calculated. Through empirical tests, the best parameters for our SVM classifier were Gaussian kernel function with 0.35 for kernel scale.

Classification accuracy was defined as

(3) $$\begin{array}{c} \text{accuracy}=\frac{\text{TP}+\text{TN}}{\text{TP}+\text{TN}+\text{FP}+\text{FN}}, \end{array}$$

where TP = number of true positives, TN = number of true negatives, FP = number of false positives, and FN = number of false negatives.

Success rate was defined as

(4) $$\begin{array}{c} \text{success}\;\;\mathrm{rate}=\frac{\sum^{}R_{\text{TP}}}{v\tau }, \end{array}$$

where *R*_TP_ is the true positive rate, *v* indicates the number of preprocessing methods, and *τ* represents the number of tasks.

Cross validation is a statistical method for assessing how the result models will generalize to an unknown dataset [38]. In this research was used LOO cross validation, where the number of folds equals the number of samples in the dataset. Thus, the SVM algorithm was applied once for each sample, using all other samples as a training set and using the selected samples as a single-item test set. As we have three classes (i.e., *S*_H_, *S*_PD,_ and *S*_DBS_), it was employed a multiclass classification [39] in a one-versus-all strategy, which employs binary classifiers to assume that one class is positive and the rest are negative.

## 3. Results

The experimental results of the assessed classification methods are shown in this section.

One hundred and seventy-one training samples were collected from 38 subjects within the training set, each composed of 408 to 1,224 dimensional features, which were reduced to two-dimensional features and evaluated with leave-one-out cross validation (LOO CV). The rest of the samples, which is 10% as described in Section 2.4, compose the test set. Each data from the test set was submitted to the out-of-sample extension in order to be mapped in a 2-dimensional space. In the end, these 2D points were labeled by the SVM model.

### 3.1. Visual Representation of Mappings

**Figure 5:**
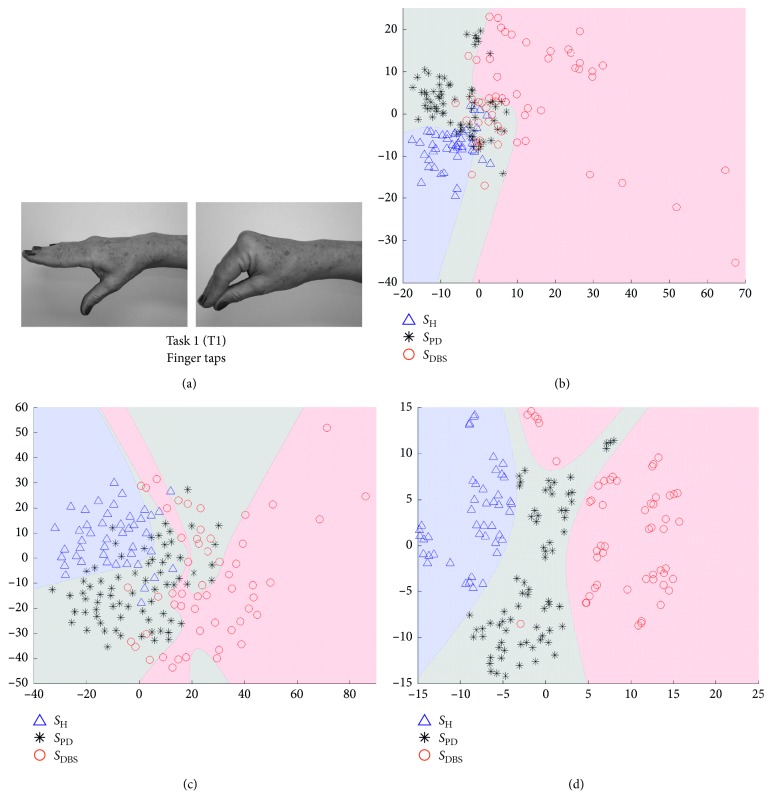
Visualization of projected data onto a lower dimensional space (step 3 in Figure 3). The visualizations are scatter plots representing dimensionless scores of the projection of high-dimensional feature vectors onto the first (*x*-axis) against the second (*y*-axis) estimated components. (a) The data are from Task 1, which is the movement of finger taps. Triangles represent *S*_H_, asterisks *S*_PD_, and circles *S*_DBS_. (b) The projections of PCA technique, (c) projections of Sammon's mapping, and (d) *t*-SNE map.

**Figure 6:**
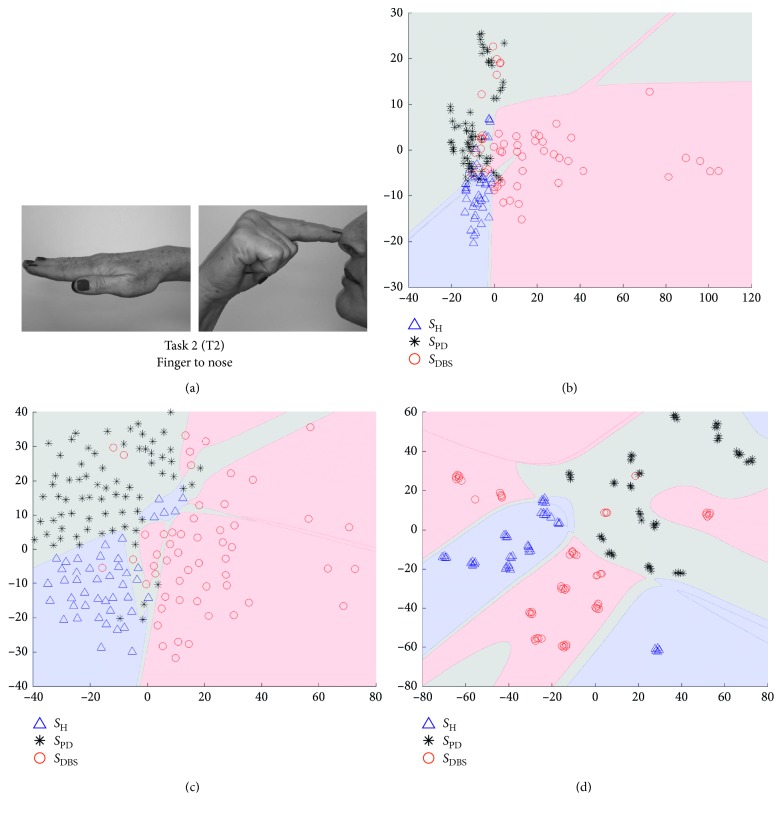
Visualization of projected data onto a lower dimensional space (step 3 in Figure 3). The visualizations are scatter plots representing dimensionless scores of the projection of high-dimensional feature vectors onto the first (*x*-axis) against the second (*y*-axis) estimated components. (a) The data are from Task 2, which is the movement of finger taps. Triangles represent *S*_H_, asterisks *S*_PD_, and circles *S*_DBS_. (b) The projections of PCA technique, (c) projections of Sammon´s mapping, and (d) *t*-SNE map.

**Figure 7:**
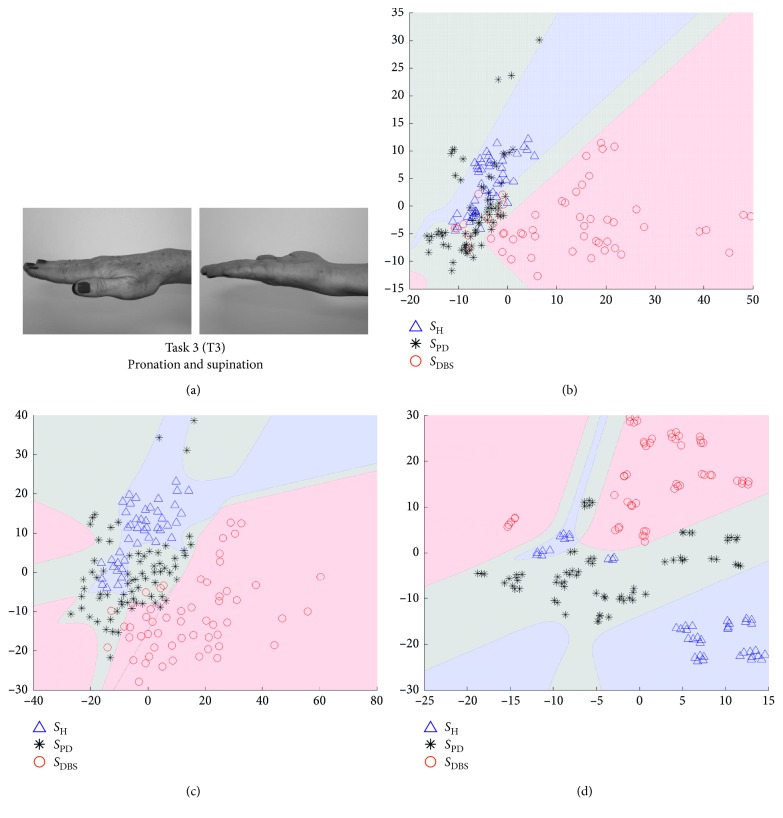
Visualization of projected data onto a lower dimensional space (step 3 in Figure 3). The visualizations are scatter plots representing dimensionless scores of the projection of high-dimensional feature vectors onto the first (*x*-axis) against the second (*y*-axis) estimated components. (a) The data are from Task 3, which is the movement of finger taps. Triangles represent *S*_H_, asterisks *S*_PD_, and circles *S*_DBS_. (b) The projections of PCA technique, (c) projections of Sammon's mapping, and (d) *t*-SNE map.

**Figure 8:**
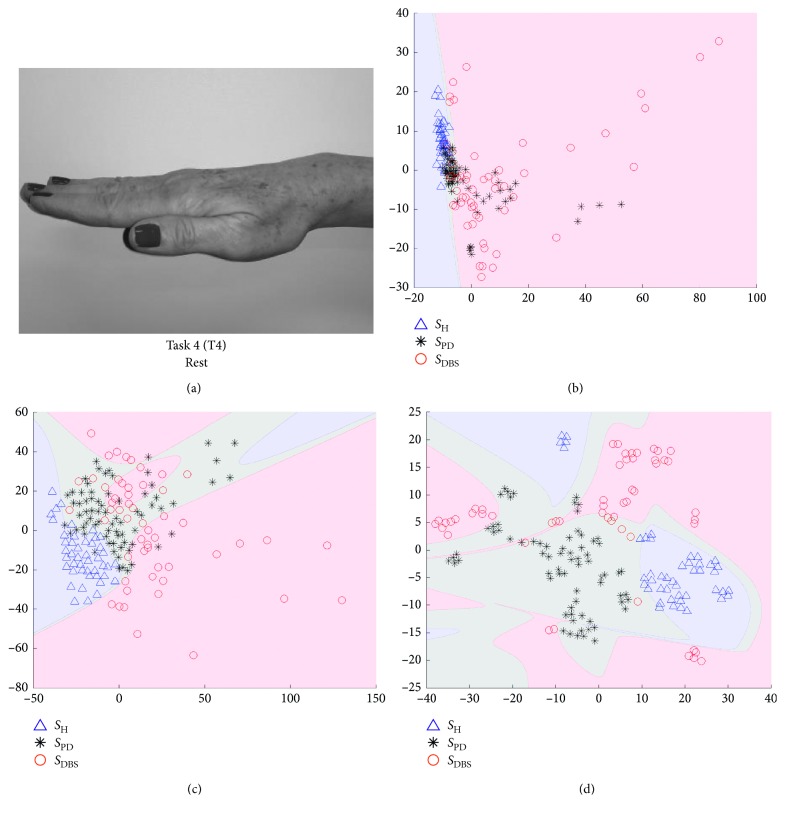
Visualization of projected data onto a lower dimensional space (step 3 in Figure 3). The visualizations are scatter plots representing dimensionless scores of the projection of high-dimensional feature vectors onto the first (*x*-axis) against the second (*y*-axis) estimated components. (a) The data are from Task 4, which is the movement of finger taps. Triangles represent *S*_H_, asterisks *S*_PD_, and circles *S*_DBS_. (b) The projections of PCA technique, (c) projections of Sammon's mapping, and (d) *t*-SNE map.

In Figures 5–8, we show some of the results of our experiments with PCA, Sammon's mapping, and *t*-SNE on the datasets built with the tasks depicted in Figure 1. The visualizations are scatter plots representing dimensionless scores of the projection of high-dimensional feature vectors. Additionally, it was drawn the decision boundary generated by a multilayer feedforward network in such a way to enhance the visual analysis.

Each setup, as depicted in Figure 4, creates one scatter plot. The scatter plots shown in Figures 5–8 were selected using a quality ratio defined as

(5) $$\begin{array}{c} \text{QR}=\frac{\left({\text{OSR}}_{\text{LOOCV}\;\;}+{\text{OSR}}_{\text{TS}\;\;}\right)}{2}, \end{array}$$

where OSR is the overall success ratio defined by

(6) $$\begin{array}{c} E=\frac{{\sum \mathrm{TP}}^{}}{\text{TNS}}, \end{array}$$

where TP is the number of true positive of all classes and TNS is the total number of samples. Since OSR is given in percentage and could range from 0 to 100%, QR also follows this interval.

This ratio aims to guide in the selection of scatter plots which reach best results in the classification process, considering each DR method and each task. In this way, Figures 5–8 represent the scenarios which achieved higher quality ratio.  Table 2 summarizes the parameters and performance values for each selected scenario.

Analyzing Table 2, *t*-SNE achieved better performance in all scenarios, reaching mean QR of 99.42%. Secondly it was Sammon's mapping with mean QR of 90.72% and finally PCA with mean QR of 81.36%. Finger to nose (T2) was the task with highest QR value considering all DR methods, and Rest (T4) was the task with the lowest performance.

### 3.2. Classification Performance of Projected Data

**Figure 9:**
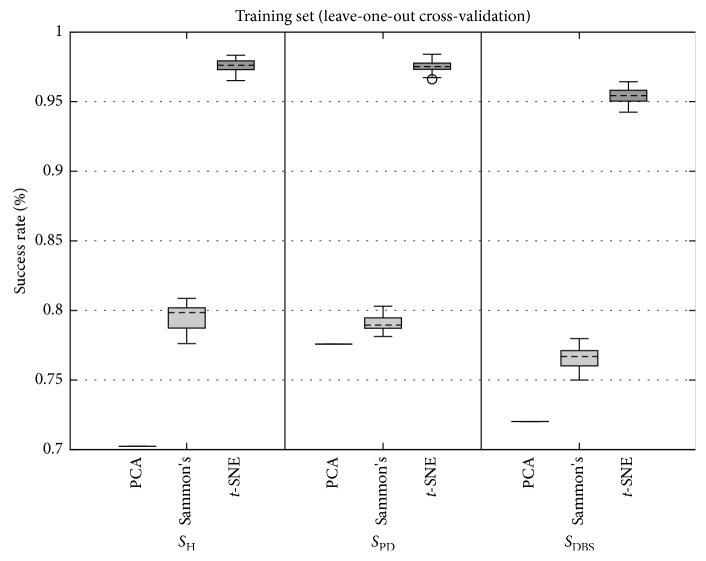
Boxplots of grand average of true positive success rate achieved by SVM using LOOCV for PCA, Sammon's, and *t*-SNE DR techniques for participants of *S*_H_ (left), *S*_PD_ (center), and *S*_DBS_ (right) groups. As in this study, PCA has no parameters to be varied (Figure 4); it is depicted by one value, which represents all possible combinations for the PCA DR method.

**Figure 10:**
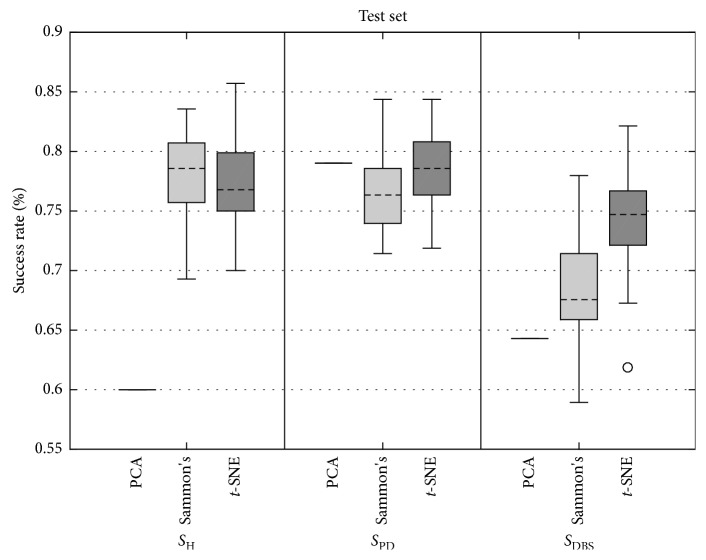
Boxplots of grand average of true positive success rate achieved by SVM using the test set for PCA, Sammon's, and *t*-SNE DR techniques for participants of *S*_H_ (left), *S*_PD_ (center), and *S*_DBS_ (right) groups. As in this study, PCA has no parameters to be set (Figure 4), it is depicted by one value, which represents all possible combinations for the PCA DR method.

Figures 9 and 10 present the boxplots of success rate (normalized between 0 and 1, in which 1 means 100%) for the data from the training set and test set, respectively. In Figure 9, for all three classes of data, the true positive success rate distribution remains similar, except for PCA for the *S*_PD_ class. In Figure 10, the true positive success rate of Sammon's mapping and *t*-SNE were similar and higher than PCA for *S*_H_ class. For *S*_PD_ and *S*_DBS_ classes, *t*-SNE yielded superior performance.

Analyzing the boxplots of Figure 9, it is observed that there is a clear difference among all DR methods, whereas in Figure 10 for *S*_DBS_ group, there also was a difference among DR methods, but for *S*_H_ and *S*_PD_ groups, the difference was not clear.

In order to confirm the analysis of boxplots, a statistical test was conducted. Only Sammon's mapping and *t*-SNE were considered for statistical analysis since the PCA method has one value in the context of boxplots. The normality presupposition was not satisfied for any of the distributions. The normality presupposition was verified by means of the one-sample Kolmogorov–Smirnov test.  Table 3 presents the *p* values estimated by means of the two-sample Kolmogorov–Smirnov test between success ratios achieved by Sammon's and *t*-SNE methods. The statistical difference of 95% was confirmed for all cases, except for *S*_H_ group from the test set.

Overall, these findings show that when *t*-SNE is combined with either the SVM algorithm, a notable improvement is seen over other investigated DR methods. When examining the mean of each distribution shown in Figure 9, the improved classification was seen when compared *t*-SNE to Sammon's, increased 18.1%, 18.4%, and 18.8% for classes *S*_H_, *S*_PD,_ and *S*_DBS_, respectively. When examining the mean of each distribution shown in Figure 10, the improved classification was seen when compared *t*-SNE to Sammon's, increased 2% and 6% for classes *S*_PD_ and *S*_DBS_, respectively, but decreased by 0.6% for class *S*_H_.

Next, Table 4 shows the grand average confusion matrix of SVM classifier for all studied DR methods, including data from the training set (LOOCV) and test set. In this table, the diagonal cells in bold show the normalized percentage of correct classifications by the SVM. For example, 70 samples of *S*_PD_ group were correctly classified when *t*-SNE DR method was employed. This corresponds to 98% of all training set samples of *S*_PD_ group. Similarly, 6 samples of the same group were correctly classified when, again, *t*-SNE DR method was employed. This corresponds to 78% of the test set samples of *S*_PD_ group.

Overall, using the PCA DR method 73.5% of the training set and 67.8% of the test set was correctly classified. For Sammon's mapping, considering the training and test sets, respectively, 78.6% and 74.1% of the predictions were correct. Lastly, *t*-SNE yielded the highest percentage of correct predictions for both, training (96.9%) and test sets (76.6%).

**Figure 11:**
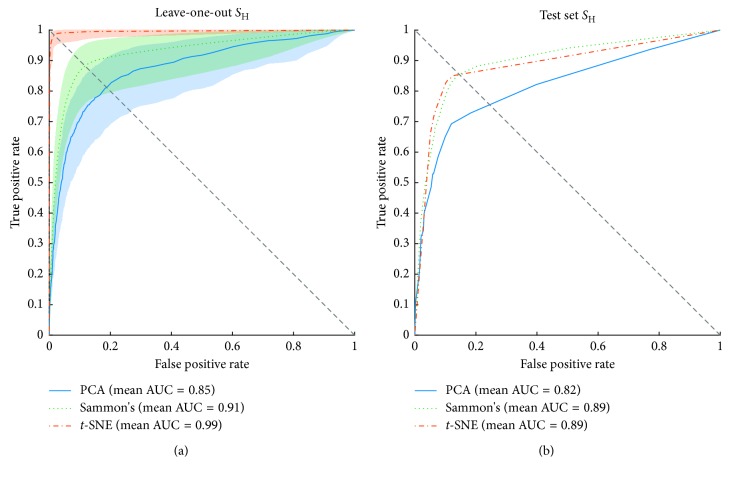
Receiver operating characteristics (ROC) curves of SVM classifier for the training and test sets of *S*_H_ group. The orange, green, and blue lines show the ROC curves for *t*-SNE, Sammon's mapping, and PCA DR methods, respectively. AUC is the area under the curve. (a) Mean ROC curves for data from the training set and its 95% confidence bounds computed by means of Bootstrap, with 1,000 replicas. (b) Mean ROC curves for data from the test set.

**Figure 12:**
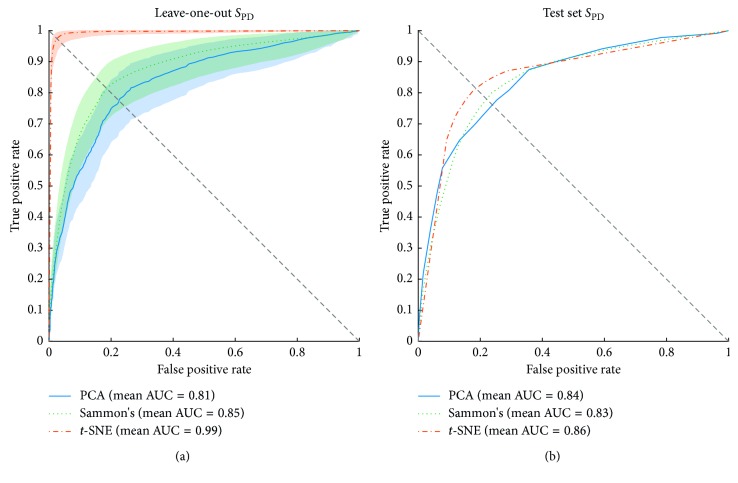
Receiver operating characteristic (ROC) curves of SVM classifier for the training and test sets of *S*_PD_ group. The orange, green, and blue lines show the ROC curves for *t*-SNE, Sammon's mapping, and PCA DR methods, respectively. AUC is the area under the curve. (a) Mean ROC curves for data from the training set and its 95% confidence bounds computed by means of Bootstrap, with 1000 replicas. (b) Mean ROC curves for data from the test set.

**Figure 13:**
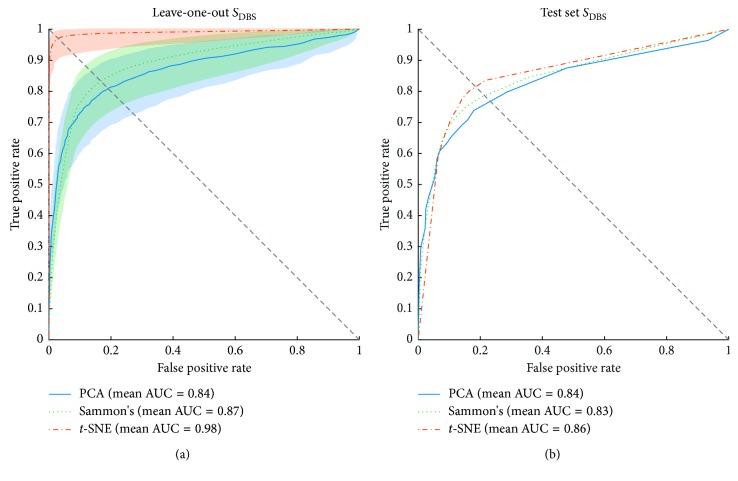
Receiver operating characteristics (ROC) curves of SVM classifier for the training and test sets of *S*_DBS_ group. The orange, green, and blue lines show the ROC curves for *t*-SNE, Sammon's mapping, and PCA DR methods, respectively. AUC is the area under the curve. (a) Mean ROC curves for data from the training set and its 95% confidence bounds computed by means of Bootstrap, with 1000 replicas. (b) Mean ROC curves for data from the test set.

Figures 11–13 show the ROC curves of the LOOCV of the training set and test set validations for each class along with the mean area under the curve (AUC) while each DR method was employed as a step before classification process. For the LOOCV, the confidence bounds of 95% were computed for ROC curves by means of Bootstrap, with 1,000 replicas.

For the *S*_H_ class, *t*-SNE achieved remarkable performance considering LOOCV, with the highest mean AUC (0.99) and with the lowest deviation from the mean. Sammon's mapping and PCA reached mean AUC of 0.91 and 0.85, respectively, and both showed a similar deviation from the mean. Considering the test set, *t*-SNE and Sammon's mapping show similar responses when observing the shape of the curve, mean AUC, and the balance point (i.e., the point where the ROC curve reaches the equality between specificity and sensitivity—diagonal dashed line in Figures 11–13). PCA, on the other hand, had the lowest performance.

The ROC curves of Figure 12 show the discrimination ability of the SVM classifier for *S*_PD_ class for both, training (LOOCV) and test validation sets. Examining Figure 12(a), the results indicate that *t*-SNE obtained similar results when compared to the same method applied in *S*_H_ group, whereas Sammon's and PCA decreased their performance. Note that for *S*_PD_ class, these two methods present overlapped area in ROC curve along with confidence bounds as much as for *S*_H_ class. However, for *S*_PD_ class, the confidence bounds are narrower.

Figure 12(b) shows ROC curves for the test set. The behavior of the curve for each DR method was similar, *t*-SNE reached the best AUC (0.86), right after are PCA (0.84) and Sammon's with AUC of 0.83. At the balance point view, *t*-SNE was the best method and PCA was the worst one. Considering *S*_H_ and *S*_PD_ classes, PCA improved for classification of *S*_PD_ samples from the test set. On the other hand, Sammon's and *t*-SNE decreased its performance for *S*_PD_ class.

The classification performance for *S*_DBS_ class is also shown in ROC curves of Figure 13. The results showed in Figure 13(a) present training set performance curves for *S*_DBS_ class, again, *t*-SNE achieved the best performance in terms of AUC and balance point. Next, Sammon's mapping and PCA with 0.87 and 0.84 of mean AUC, respectively, showing great overlapped area between its confidence bounds. For *S*_DBS_ class, *t*-SNE showed the wider confidence bound while compared with the performance achieved for *S*_H_ and *S*_PD_ classes.  Figure 13(b), in turn, shows that the three DR methods yielded the same results for *S*_PD_ and *S*_DBS_ classes in terms of mean AUC.

## 4. Discussion

This kind of study is not often found in the literature. The reasons could be related to the complexity of the recruitment of volunteers since, in this study, three distinct groups (i.e., *S*_H_, *S*_PD,_ and *S*_DBS_) were evaluated. This type of data are expensive, and their acquisition demands specialized professionals.

In the literature, there are a plenty of studies which propose and evaluate methods for discrimination between individuals with PD from neurologically healthy ones. However, some studies show that there are key points to be overcome for realizing the full potential of this technology in PD research and practice [40, 41], for instance (1) the machine learning methods are challenging to evaluate and apply without a basic understanding of the underlying logic on which they are based; (2) the ability to algorithmically analyze and synthetically display clinically and disease-relevant information to physicians and patients remains limited. This study brings a comparison among three DR methods with the aim to address these two points.

PD treatment is also another topic extensively discussed. The two fields inside this area related to our study are an investigation of motor behavior while using medication-based treatments and surgical ones. According to [40], it is lacking an objective way to adjust drug (e.g., levodopa) release as the patient needs. Besides that, the DBS treatment has different points for improvements, one of that concerns the implementation of closed-loop (i.e., self-adjustable parameters) DBS. The present study moves toward these directions, comparing these groups of subjects and characterizing its motor behavior.

As reported in the literature [14, 20, 42–44], our results demonstrated differences between movement patterns for the three groups. On the other hand, we introduce the comparison of visualization and classification tools, which allows for an objective evaluation of subjects. Based on our review, just a few studies approached the challenge of visualizing and classifying motor activities of the three classes evaluated. Even so, the studies that explored this area did not go as far as our study.

The visual representation of mappings presented in Figures 5–8 show the ability of each DR technique to deal with high-dimensional data since these figures show the scenarios which achieved higher quality ratio. Considering the visual aspect (i.e., clustering and boundary of classes), *t*-SNE produces better visualizations, followed by Sammon's mapping in second place and PCA in the third one. In fact, the *t*-SNE ability to keep global and local structures implies in better visualizations as stated in [25]. Sammon's mapping, in turn, improves PCA, adding the ability to handle with nonlinear data. In every mentioned figure, the map built by Sammon's has a similar shape while compared with PCA map. This occurs due to the PCA initialization strategy for Sammon's algorithm [45].

Classification accuracy for PCA, Sammon's mapping, and *t*-SNE was, respectively, 73.5%, 78.6%, and 96.9% for the training set and 67.8%, 74.1%, and 76.6% for the test set. According to [38], the training set is used to fit the models and the test set is used for assessment of the generalization error of the final chosen model. Furthermore, there are subtle differences between the training set and test set. The reasons of that are (1) differences in motor behavior between inter and intragroups; (2) the training and test sets are built randomly; (3) the out-of-sample step introduces error which is related to the mapping of high-dimensional information onto a 2-dimensional space; (4) the classifier generalization ability varies, and this factor impacts directly in the prediction accuracy, especially when new samples are presented.

Visual representation presented in Figures 5–8 could be used as a visualization tool for follow-up of treatments of PD by means of definition of the control zone, so that the closer this zone to the subject is better in terms of motor behavior. Furthermore, to achieve a smooth control of this zone, an individual analysis for each patient could help.

Our results take into account the differentiation of PD treatments and a healthy control group without considering the subtypes of the disease. The variability found in some methods may be due to this factor, since tremor, bradykinesia, and rigidity present different movement patterns. A further study with the use of our system and protocol in new groups of participants, separated by PD subtypes, could address this limitation.

The tasks performed in this study are well established, described in the UPDRS [46] and used in clinical evaluation [47–52]. In Figure 5, the finger taps (Task 1) using *t*-SNE projection reached a quality ratio of 99.42% ± 0.8 as well as the clearer visual representation among all mappings shown in Figures 5–8. Sammon's mapping, in turn, presented a spherical projection, which is characteristic of this method and achieved 88.60% ± 8.6 of QR, around 10% less than *t*-SNE.

Finger to nose (T2) and pronation and supination (T3) were the performed tasks with highest mean QR, 93.47% and 92.50%, respectively, considering all DR methods. Both movements are more complex than the other two performed tasks, finger taps (T1), and rest (T4). The higher motor pattern complexity of T2 and T3 tasks reflect in a higher success rate on discrimination of the three classes (*S*_H_, *S*_PD_, and *S*_DBS_). The finger to nose task shares its dominant kinematic pattern with a variety of activities of daily living (ADL) such as eating, drinking, and answering a phone. Pronation and supination task, on the other hand, is a commonly used task to assess bradykinesia [53, 54].

Regarding discrimination among groups, *t*-SNE showed the highest success rates for the LOOCV followed by Sammon's mapping. Similar performance was achieved when *t*-SNE was applied as a step before proceeding with the classification using the test set. Although the success rate reached by *t*-SNE was superior, its performance was weak while compared with itself in LOOCV. This drop occurs due to the step to allow project new data points, called out-of-sample (step 4 in Figure 3). The out-of-sample (OOS) process was carried out by means of a PCA along with an ANN as explained in Section 2.6. Our OOS approach reached overall mean squared error of 17.9 ± 10.5 and 3.6 ± 2.7 for Sammon's mapping and *t*-SNE, respectively, and an overall R value of 0.97 ± 0.02 and 0.95 ± 0.03 also for Sammon's mapping and *t*-SNE, respectively.

Despite our good results in OOS step, in many cases, the high variability of intragroup motor patterns, mainly in *S*_PD_ and *S*_DBS_, turns the OOS a hard process. There are in the literature other methods to deal with OOS [55]; these methods could improve the results presented in this study.

In this study, three preprocessing methods were employed. The first (FS) was based on the filtered signal, which yields data more correlated with the original data; the second (IF) captures changes in the signal frequency over time and the third (IA) takes into account changes in the amplitude of the signal.

Concerning to the preprocessing methods, our results show that the combination of features extracted from the methods FS and IF was the one that yielded the best overall success rate (86.14% ± 4.3), in accordance with [20]. The success of this combination may be related to the cardinal symptom tremor, which induces oscillatory movements in individuals with PD. These oscillatory movements could vary around 6 Hz [52].

Proceeding to classification analysis, Table 4 summarizes the classification results by using the confusion matrix style. Machado et al. [20] employed a similar analysis in some points, using only Sammon's mapping. They reported an overall mean success rate as given below:

- *S*_H_ (*S*_H_): 0.85 and 0.75 for classification and test sets, respectively;
- *S*_PD_ (*S*_PD_): 0.73 and 0.60 for classification and test sets, respectively;
- *S*_DBS_ (*S*_DBS_): 0.72 and 0.63 for classification and test sets, respectively.

In our experiments using *t*-SNE we achieved an overall mean success rate as given below (from Table 4):

- *S*_H_ (*S*_H_): 0.98 and 0.77 for classification and test sets, respectively;
- *S*_PD_ (*S*_PD_): 0.98 and 0.78 for classification and test sets, respectively;
- *S*_DBS_ (*S*_DBS_): 0.95 and 0.74 for classification and test sets, respectively.

## 5. Conclusion

This study investigated the motor behavior of three distinct groups of individuals: neurologically healthy, PD treated with levodopa, and PD treated with DBS. In order to analyze the motor behavior of each group, four motor tasks were performed by the subjects and recorded using inertial and EMG sensors. In spite of the large possibilities of sensors to be used for collecting various data that can quantify PD symptoms, the same progress cannot be seen while dealing with large and complex data such as the kind of data collected in this study.

The assessment of the classification methods showed that the visualization provided by the *t*-SNE enhanced the visual discrimination of the groups so that they could be clearly identified for all investigated tasks. For automatic discrimination among groups, SVM was used after the data reduction step. The SVM performance was higher in almost all scenarios while *t*-SNE was employed. Furthermore, the noted improvement was irrespective of the group or task or of the preprocessing method utilized, with an improvement of around 18% for the training set, considering *t*-SNE versus Sammon's mapping. For *t*-SNE versus PCA, the improvement was around 23% for the training set.

## Figures and Tables

**Table 1:** Overall mean coefficient of variation (1 = 100%) among each repetition of the subject.

Coefficient of variation per group		
*S*_H_	*S*_PD_	*S*_DBS_
0.21 ± 0.14	0.24 ± 0.15	0.30 ± 0.21

**Table 2:** Parameters and performance values of best scenarios according to its quality ratio (QR).

Task	DR methods	Parameter settings			Performance values
		*l*	*η*	*ε*	QR (%)
Finger taps (T1)	PCA	−	−	−	79.82 ± 6.2
	Sammon's	575	0.6	−	88.60 ± 8.6
	*t*-SNE	5000	0.6	27	99.42 ± 0.8

Finger to nose (T2)	PCA	−	−	−	86.26 ± 4.5
	Sammon's	423	0.4	−	94.44 ± 7.8
	*t*-SNE	5000	0.4	5	99.71 ± 0.4

Pronation and supination (T3)	PCA	−	−	−	83.92 ± 15.2
	Sammon's	742	0.6	−	93.57 ± 9.0
	*t*-SNE	1000	0.6	16	100 ± 0.0

Rest (T4)	PCA	−	−	−	75.44 ± 2.4
	Sammon's	1080	0.4	−	86.26 ± 11.9
	*t*-SNE	3000	0.5	16	98.54 ± 2.0

**Table 3:** *P* value from two-sample Kolmogorov–Smirnov test between success ratios achieved by Sammon's and *t*-SNE methods.

Group	*P* value	
	Leave-one-out cross validation	Test set
*S*_H_	0.00	0.32
*S*_PD_	0.00	0.02
*S*_DBS_	0.00	0.00

**Table 4:** Grand average confusion matrix of SVM for each DR method. The bold diagonal cells show the normalized (0–1) percentage of correct classifications by the SVM.

DR method			Target class					
			Leave-one-out cross validation			Test set		
			*S*_H_	*S*_PD_	*S*_DBS_	*S*_H_	*S*_PD_	*S*_DBS_
PCA	Predicted class	*S*_H_	**0.70**	0.11	0.07	**0.60**	0.08	0.10
		*S*_PD_	0.24	**0.78**	0.20	0.31	**0.79**	0.26
		*S*_DBS_	0.05	0.11	**0.72**	0.09	0.13	**0.64**

Sammon's	Predicted class	*S*_H_	**0.79**	0.08	0.05	**0.78**	0.11	0.08
		*S*_PD_	0.15	**0.79**	0.18	0.17	**0.76**	0.24
		*S*_DBS_	0.05	0.13	**0.77**	0.06	0.13	**0.68**

*t*-SNE	Predicted class	*S*_H_	**0.98**	0.01	0.01	**0.77**	0.08	0.07
		*S*_PD_	0.02	**0.98**	0.03	0.14	**0.78**	0.19
		*S*_DBS_	0.01	0.01	**0.95**	0.09	0.14	**0.74**

## Data Availability

The set of features used to support the findings of this study are included in the supplementary information in a Microsoft Excel file.

## Supplementary Materials

Supplementary Materials The attached supplementary material file includes three tables containing features estimated from signals of the executed tasks. Each table includes features extracted from filtered signals, instantaneous frequency, and instantaneous amplitude, respectively.
